# A childhood chemotherapy protocol improves overall survival among adults with T-lymphoblastic lymphoma

**DOI:** 10.18632/oncotarget.9144

**Published:** 2016-05-02

**Authors:** Meng-yuan Zhu, Hua Wang, Chun-yu Huang, Zhong-jun Xia, Xiao-qin Chen, Qi-rong Geng, Wei-da Wang, Liang Wang, Yue Lu

**Affiliations:** ^1^ State Key Laboratory of Oncology in South China, Sun Yat-Sen University Cancer Center, 510060 Guangzhou, Guangdong, P.R. China; ^2^ Department of Hematological Oncology, Sun Yat-Sen University Cancer Center, 510060 Guangzhou, Guangdong, P.R. China; ^3^ Department of Endoscopy, Sun Yat-Sen University Cancer Center, 510060 Guangzhou, Guangdong, P.R. China; ^4^ Collaborative Innovation Center of Cancer Medicine, Sun Yat-Sen University Cancer Center, 510060 Guangzhou, Guangdong, P.R. China

**Keywords:** T-lymphoblastic lymphoma, treatment, adult, childhood, chemotherapy

## Abstract

A broadly accepted standard treatment for adult T-lymphoblastic lymphoma (T-LBL) has not yet been defined. To address that issue, we retrospectively compared three chemotherapy regimens used to treat 110 adult patients with newly diagnosed T-LBL. These included two adult regimens (ECOG2993 and hyper-CVAD) and a childhood regimen (BFM-90). These intensive drug regimens are mainly used to treat childhood and adult acute lymphoblastic leukemia. They included induction, consolidation, and maintenance chemotherapy protocols and were administered over the course of 2 years. Seventy-five patients (80%) achieved a complete remission (CR). Within a median follow-up time of 31 months (range: 5–152 months), the 5-year overall survival (OS) and progression-free survival (PFS) rates were 47.7% (95% CI, 35.0–69.8%) and 45.7% (95% CI, 27.6–56.6%), respectively. Shorter survival was associated with age > 40 years, poor ECOG PS and bone marrow involvement. Elevated lactic dehydrogenase (LDH) level, Ann Arbor stage and International Prognostic Index (IPI) score had no prognostic value. The childhood chemotherapy regimen improved CR and the overall survival rate more than the adult regimen in patients aged < 40 years.

## INTRODUCTION

T-lymphoblastic lymphoma (T-LBL) represents less than 2% of adult non-Hodgkin lymphomas (NHLs), but accounts for 85% to 90% of all lymphoblastic lymphomas [1]. This high-grade lymphoma occurs mostly in males, with a high incidence of mediastinal tumors and several distinct clinical signs, including cough, shortness of breath, respiratory distress, and/or superior vena cava (SVC) syndrome [1]. At one time, LBL and acute lymphoblastic leukemia (ALL) were considered to be the same disease due to the similarity of their biological characteristics. However, the two diseases have different clinical presentations and treatment responses. No reliable risk factors or prognostic factors have been identified for adult patients with T-LBL, though the impact of several potential risk factors, including B-phenotype, elevated LDH, IPI and central nervous system (CNS) involvement, has varied among trials [2, 3]. Cytogenetic abnormalities show no prognostic value in adult, though they are related to an aggressive clinic course. No chromosomal or molecular abnormalities have been consistently shown to carry prognostic significance except t (9;17) (q34;3), which is associated with an aggressive clinical course in children [4, 5].

At our cancer center, doctors have treated LBL patients using several different chemotherapy regimens, including the hyper-CVAD, ECOG E2993 and BFM-90 protocols. The purpose of this retrospective study was to further analyze the clinical characteristics and biological and prognostic factors in T-LBL, and to evaluate the regimens used to treat adult (hyper-CVAD and ECOG E2993) and childhood (BFM-90) T-LBL. We then sought to determine which patients benefit from the adult or childhood regimen, as this could be applied to guide us to devise more personalized treatments.

## RESULTS

### Patient characteristics

The main clinical characteristics of the 110 patients are presented in Table 1. The median age was 28 years (range: 18–65 years) with 80% under 40 years. The male:female ratio was 7:3. Eighty-three patients (75%) had a good ECOG PS of 0–1. Using the Ann Arbor staging system, 90 patients (81%) presented with stage III or IV disease. B symptoms were reported in 43 patients (39%) at diagnosis. Nighty-three patients (85%) had a mediastinal mass. Bone marrow (BM) involvement was common, occurring in 55% of all patients. LDH levels were frequently elevated. Based on IPI scores, more than half of the patients had low or low-to-moderate risk disease. The comparative baseline characteristics of all patients with respect to administration of childhood and adult regimens are summarized in Supplementary Table 1. There were no significant differences in the frequency of these characteristics between the two treatment regimens.

**Table 1:** Baseline characteristics of patients with T-LBL

Clinical Characteristics	*n* (%)
Age [median (range), years]	28 (18–65)
< 40	88 (80)
≥ 40	22 (20)
Gender	
Male	77 (70)
Femal	33 (30)
ECOG PS	
≤ 1	83 (75)
≥ 2	27 (25)
Ann Arbor stage	
I	4 (4)
II	16 (15)
III	15 (13)
IV	75 (68)
B symptoms	
Present	43 (39)
Absent	67 (61)
Mediastinal mass	
Present	93 (85)
Absent	17 (15)
Bone marrow involvement	
Positive	61 (55)
Negative	49 (45)
CNS involvement	
Positive	2 (2)
Negative	108 (98)
Median LDH level, U/L (range)	220.8 (91–7706.5)
≤ 245	54 (49)
> 245	42 (38)
NA	14 (13)
IPI	
≤ 1	59 (54)
≥ 2	37 (33)
NA	14 (13)

ECOG PS, Eastern Cooperative Group performance status; CNS, central nervous system; LDH, lactatedehydrogenase; ULN, upper limit of normal; NA, not available; B symptoms, tumor fever higher than 38°C, night sweats, and/or weight loss more than 10%; T-LBL, T-lymphoblastic lymphoma.

### Treatment response and survival

**Figure 1:**
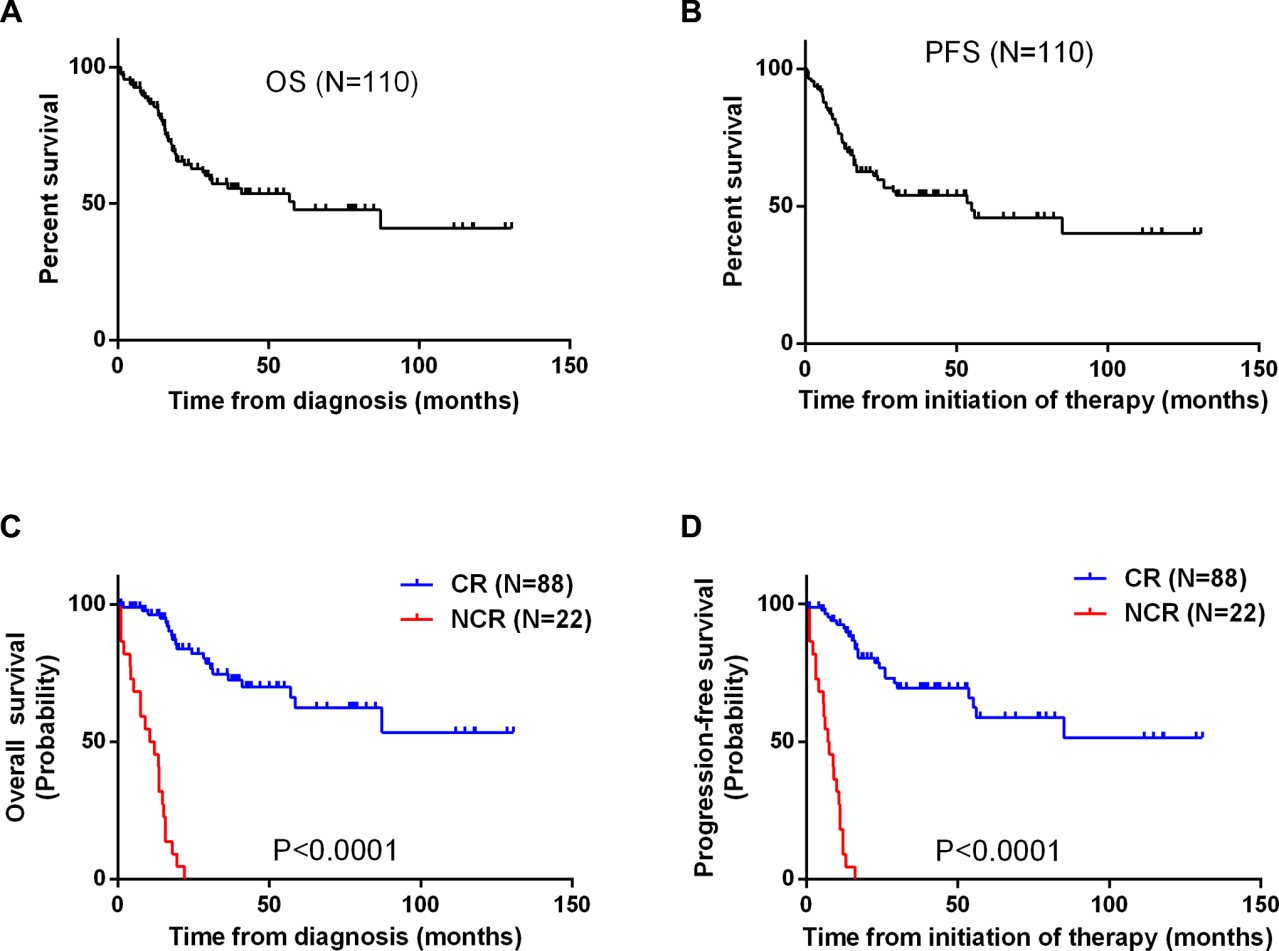
(**A**, **B**) Kaplan-Meier curves for analysis of OS (A) and PFS (B) among 110 patients with T-LBL. (**C**, **D**) Kaplan-Meier curves comparing OS (C) and PFS (D) between patients who achieved a complete response (CR) after two cycles of chemotherapy and those who did not (NCR).

Within a median follow-up time of 31months (range: 5–152 months), the 5-year OS and PFS rates for all patients were 47.7% (95% CI, 35.0–69.8%) and 45.7% (95% CI, 27.6–56.6%), respectively (Figure 1A and 1B). Eighty percent of these LBL patients achieved CR, while 15.5% achieved PR. Patients who achieved CR after two cycles of chemotherapy had a significantly better PFS and OS than those without CR (Figure 1C and 1D).

**Figure 2:**
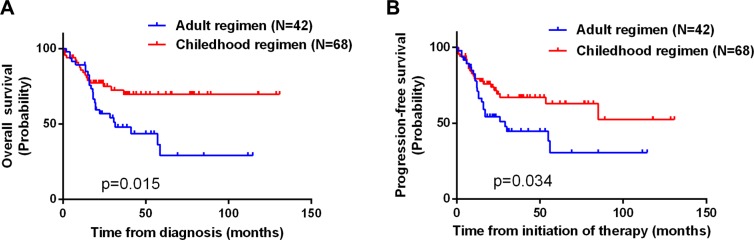
Kaplan-Meier curves illustrating the significant differences in OS (A) and PFS (B) between patients receiving an adult chemotherapy regimen and those receiving the childhood regimen (*P* = 0.015 and 0.034, respectively)

**Figure 3:**
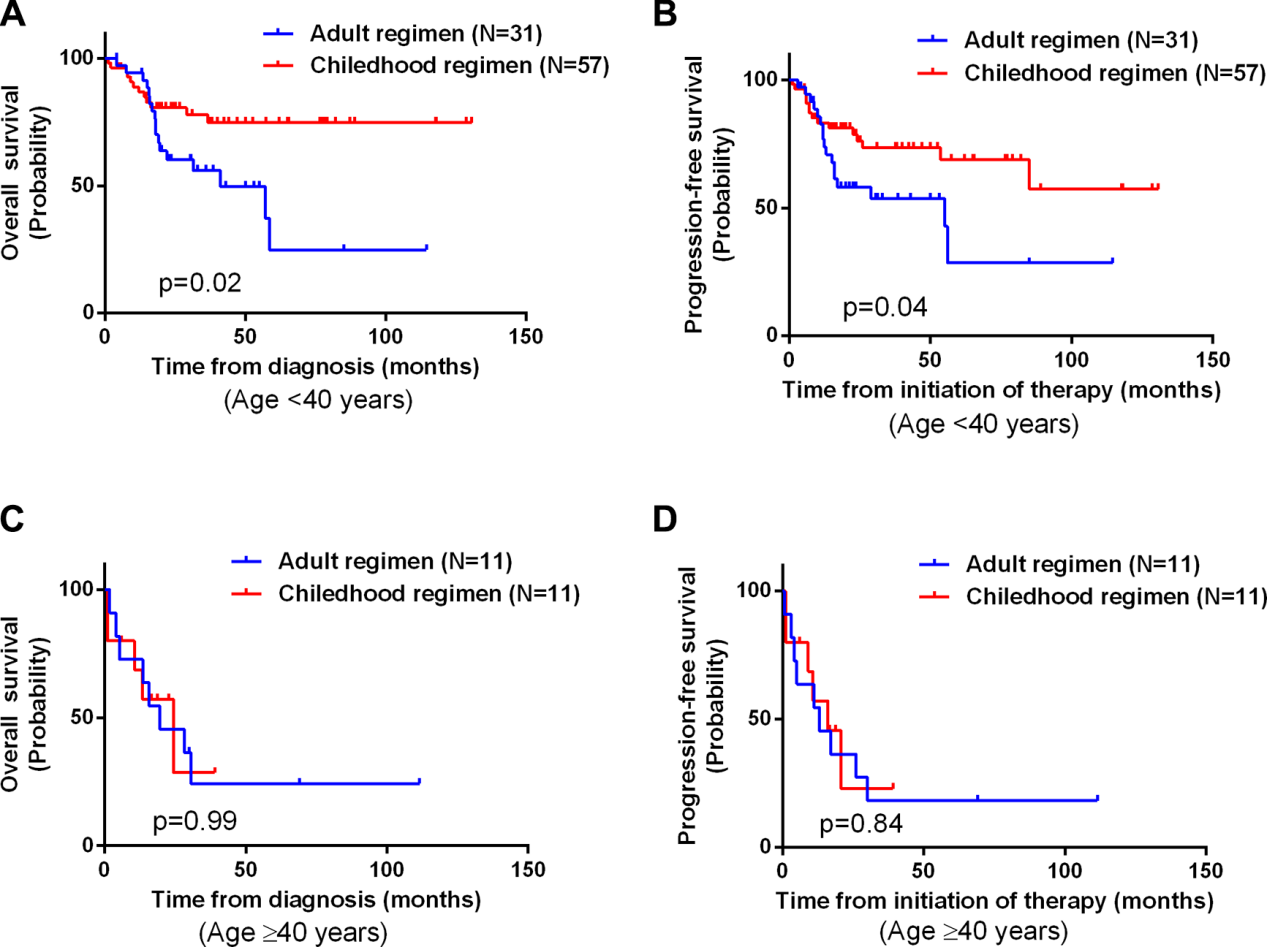
Kaplan-Meier curves comparing OS and PFS between patients receiving an adult chemotherapy regimen and those receiving a childhood regimen after dividing the patients into different age subgroups (**A**) OS of patients aged < 40 years (*P* = 0.02). (**B**) PFS among patients aged < 40 years (*P* = 0.04). (**C**) OS among patients aged ≥ 40 years (*P* = 0.99). (**D**) PFS among patients aged ≥ 40 years (*P* = 0.84).

All patients received intensive treatment regimens, including the childhood regimen BFM 90 (68 patients) and two adult regimens, ECOG E2993 (14 patients) and hyper-CVAD (28 patients). Responses to treatment are summarized in Table 2. There were no significant differences in the response rates among the three treatment regimens. On the other hand, significant differences in the 5-year OS and PFS rates were observed (OS, *p* = 0.015; PFS, *p* = 0.034). Among all patients receiving the childhood regimen, the OS rate was 62.6%, but was 38.6% among those receiving an adult regimen (Figure 2A and 2B). Subgroup analysis showed that among patients younger than 40 years, the 5-year OS and PFS rates (OS, *p* = 0.02;PFS, *p* = 0.04) for the adult regimens was again inferior to those for the childhood regimen (Figure 3A and 3B). On the other hand, the survival outcomes for the adult and childhood regimens were similar among patients aged ≥ 40 years (Figure 3C and 3D). Five patients (4.5%) who died early during induction had persistent progressive disease. Seventeen patients had progressive disease and received second-line treatment similar to ALL chemotherapy. Among that group, only 4 patients achieved CR.

**Table 2:** Treatment outcome and response rate for all patients

Chemoregimen	CR (%)	PR	5-Years PFS Rate (%)	5-Years OS Rate (%)
ECOG 2993 (*n* = 14)	78.6	21.4	48.2	45.5
BFM 90 (*n* = 68)	83.6	8.8	67.4	62.6
Hyper CVAD (*n* = 28)	71.4	28.6	36.4	31.8
Total (*n* =110)	80.0	15.5	47.7	45.7

Abbreviations: CR, complete response; PR, partial remission; PFS, progression-free survival; OS, overall survival.

We also analyzed the long survival outcomes of 27 patients who relapsed after a median of 13 months (range: 8.8–56 months). Among this group, 11 presented with recurrence in the mediastinum, four with recurrence in the BM, and one with lymph node recurrence. Eleven patients showed recurrence in both the mediastinum and BM. All of these patients had mediastinal involvement at diagnosis but never received mediastinal irradiation. No CNS recurrence was observed in this study. The relapsed patients were treated with various intensive chemotherapy regimens, and four achieved a second CR. Among the 27 relapsed patients, eight underwent high dose chemotherapy with autologous stem cell transplantation, and seven received allogeneic BM transplantation. Three patients are in continuing CR after allogeneic BM transplantation.

### Univariate and multivariate analyses

Univariate analysis showed that receiving adult regimen chemotherapy, age, ECOG PS, mediastinal mass and BM involvement were all significantly predictive of shorter OS and PFS (Table 3). No other factors were predictive of outcome. Clinical factors that were statistically significant predictors of OS in univariate analysis were included in a multivariate analysis, which revealed that receiving adult regimen chemotherapy, age, ECOG PS and BM involvement were independent prognostic factors for PFS and OS (Table 3). However, mediastinal mass failed to be prognostic under multivariate analysis.

**Table 3:** Univariate and multivariate analysis of factors associated with overall survival and progression-free survival of all patients

Clinical Characteristics	Overall Survival			Progression-Free Survival		
	Univariate analysis	Multivariate analysis		Univariate analysis	Multivariate analysis	
	*P*-value	RR (95% CI)	*P*-value	*P*-value	RR (95% CI)	*P*-value
**Chemotherapy regimen**	0.015	0.433	0.009	0.034	0.412	0.004
Childhood vs. Adult regimen		(0.232–0.809)			(0.226–0.749)	
**Age**	0.073	0.457	0.017	0.006	0.461	0.031
< 40 vs. ≥ 40		(0.241–0.8 68)			(0.301–0.947)	
**Gender**	0.503			0.358		
Male vs. Female						
**B symptoms**	0.357			0.365		
Absent vs. Present						
**ECOG PS**	0.0009	0.303	0.002	0.001	0.350	0.003
≤ 1 vs. ≥ 2		(0.144–0.635)			(0.174–0.705)	
**Mediastinal mass**	0.013			0.009		
Absent vs. Present						
**Bone marrow involvement**	0.0304	0.288	0.001	0.016	0.304	0.001
Absent vs. Present		(0.144–0.577)			(0.159–0.584)	
**LDH**	0.111			0.089		
≤ 245 vs. > 245						
**Ann Arbor stage**	0.481			0.678		
≤ 2 vs. ≥ 3						
**IPI**	0.662			0.762		
≤ 1 vs. ≥ 2						

Abbreviations: ECOG PS, Eastern Cooperative Oncology Group performance status; LDH, lactate dehydrogenase; IPI, International Prognostic Index; RR, relative risk; CI, confidence interval.

### Toxicity

All the patients exhibited III/IV grade myelosuppression during remission induction. Two patients exhibited tumor lysis syndrome. No treatment-related mortality was observed.

## DISCUSSION

The high-grade NHL protocol and the ALL chemotherapy regimen reportedly produce 5-year survival rates of less than 50% among adults with T-LBL [1, 6]. On the other hand, the German BFM group, who studied 105 children given ALL-type regimens, estimated the 5-year EFS to be 90% [7]. For T-LBL patients, it appears that the adult ALL chemotherapy regimen always produced poorer outcomes in adults than were achieved in children receiving the childhood regimen, but there were no direct comparisons between the efficacies of childhood and adult ALL regimens in adults with LBL.

This report describes the results achieved among 110 adult T-LBL patients treated with the childhood ALL regimen (BFM-90) or one of two adult regimens (ECOG E2993 or hyper-CVAD). With these approaches, a CR rate of 80% for all patients was obtained, which is comparable to the 80–90% rates previously reported with ALL regimens. The 5-year OS and PFS rates for all patients were 47.7% and 45.7%, respectively, but the childhood ALL regimen produced significantly better PFS and OS rates than the two adult regimens. Based on a Cox regression model that included multiple clinical prognostic factors, we concluded that receiving the adult regimen as chemotherapy, age, ECOG PS and BM involvement were independent prognostic factors affecting both PFS and OS.

The clinical characteristics of the T-LBL patients analyzed in the present study were similar to those previously described by others: patients were predominantly young and male with frequent mediastinal involvement [3, 8]. However, BM involvement occurred in our cohort more frequently than in other series [3, 9], while our observed absence of CNS involvement also differed from other studies. These differences may be explained by the selection of patients for this retrospective analysis after review of the pathological diagnosis.

As summarized in Table 2, the CR rates achieved with the childhood ALL regimen (BFM-90) appeared higher than those achieved with the ECOG E2993 or hyper-CVAD regimen, but the difference was not significant. The outcome with the hyper-CVAD regimen in LBL was first reported by Thomas et al. [2], while Sekimizu et al. reported that among their patients, 30 (91%) achieved CR, and 3 (9%) achieved PR [5]. But it appears that in our study patients treated with the hyper-CVAD regimen had a poorer prognosis, which may be explained by the too small sample size.

In the subgroup analysis, we found the patients aged < 40 years (Figure 3) receiving the childhood regimen had better OS and PFS rates than those receiving an adult regimen. However, the older patients did not benefit from the childhood regimen as much as from the adult regimens. This result was similar to what has been seen in ALL patients, in whom pediatric regimens were shown to be favorable for Ph negative AYA ALL patients [10–13]. Patients aged 15–40 years – i.e., adolescent and young adult (AYA) – often tolerated the side effects of more intensive ALL regimens better than adults (aged ≥ 40 years), as they had fewer underlying diseases such as hypertension, heart disease and diabetes. Studies have confirmed that the childhood ALL regimen improves survival in AYA patients with ALL because the cumulated doses of vincristine, steroids and asparaginase in the childhood ALL regimen (e.g., BFM-90) are significantly higher than in adult regimens (e.g., ECOG E2993 or hyper-CVAD). On the contrary, treatment-related toxicity partially offset the improvement brought by the dose escalation of non-cellulotoxic anticancer drugs in patients aged > 40 years [12].

Although prophylactic irradiation of the CNS was not administered to patients in the present study, no patient experienced relapse with CNS involvement after treatment. Burkhardt et al. reported that stage III/IV patients that were CNS-negative did not benefit from prophylactic cranial radiotherapy [14]. In our study, high-dose MTX in the drug regimens and regular intrathecal chemotherapy may have contributed to reducing the incidence of CNS involvement [15]. Three patients achieved continuous CR after allogeneic BM transplantation following induction therapy. The efficacy of stem cell transplantation for LBL is controversial, since chemotherapy reportedly achieves a survival rate similar [1].

Some prognostic factors that have been found to significantly affect the survival of patients with LBL include age, stage IV disease, high LDH level and anemia [3, 8, 9, 16, 17]. In the German Multicentre Trials for Adult Acute Lymphoblastic Leukemia (GMALL) series on T-LBL, the only significant prognostic factor for survival was elevated LDH [16]. In the MDACC series [2], only CNS involvement at diagnosis was significantly associated with poorer outcome. In the present study, however, neither Ann Arbor stage nor LDH level influenced OS or PFS. In addition, Hoelzer et al. [16] reported the inability of the IPI index to predict LBL outcome. Thus, a convincing prognostic model for adult T-LBL has yet to be defined. In our study, multivariate analysis revealed that age, ECOG PS and BM involvement at diagnosis are independent prognostic factors affecting OS and PFS, which is consistent with the observation reported by Morel et al. [3] that 40 years of age appears to be a cut-off for OS. In sum, our study is one of the largest conducted on adult T-LBL patients (*n* = 110) treated with different ALL-type chemotherapy regimens (ECOG E2993, hyper-CVAD and BFM-90). The childhood regimen appeared to give better OS rates than the adult regimens in patients aged < 40 years, and age, ECOG PS and BM involvement at diagnosis were independent prognosis factors affecting OS and PFS. Future prospective studies will required to confirm our findings.

## MATERIALS AND METHODS

### Patient selection

Selected for this study were 110 patients with pathologically proven adult T-LBL diagnosed between August 2000 and June 2015 at the Sun Yat-Sen University Cancer Center. The histology and immunophenotype were reviewed to confirm the diagnosis based on World Health Organization guidelines. The criteria for case inclusion were: (1) histologically confirmed diagnosis of T-LBL; (2) T-LBL cell type confirmed using immunohistochemistry or flow cytometry; (3) no previous malignancy; (4) no previous treatment for lymphoma; and (5) adequate clinical information and follow-up data. The clinical data included the following information: patient demographics, physical examination, Eastern Cooperative Oncology Group performance status (ECOG PS), B symptoms, serum LDH, BM examinations, computed tomography (CT) or magnetic resonance imaging (MRI) of the involved field, or whole body positron emission tomography/computed tomography (PET/CT). All patients were staged according to the Ann Arbors stage system [18], as calculated using the IPI [19]. BM involvement was defined as more than 5% blast cells in the BM. Patients who had more than 25% blasts in the BM were defined as T-ALL and excluded from this study. CNS involvement was evaluated using lumbar puncture at the time of diagnostic workup. CNS involvement was defined as ≥ 5 WBCs/mcl in the cerebrospinal fluid, with the presence of lymphoblasts. Both the Institutional Review Board and Ethics Committees of Sun Yat-Sen University Cancer Center approved this study. All patients consented to allow their medical records be used for research purposes.

### Treatment

The numbers of patients who received each chemotherapy regimen are summarized in Table 2. All details of the chemotherapy regimens have been reported previously [2, 7, 20]. The treatment response was assessed after every administration cycle. CR was defined as the disappearance of all clinical evidence of disease, normalization of all laboratory abnormalities related to the lymphoma, and normalization of radiographic images and biopsies that had been abnormal before therapy. PR was defined as regression of the tumor burden by > 50%. Tumor volume regression of < 50% and progressive disease were considered to be failures. After completing their treatment, patients were followed up and assessed by their oncologist in our outpatient department. Each follow-up visit consisted of a physical examination; complete blood count; serum biochemistry, including LDH levels; BM examination; and a whole body CT scan. Follow-up visits were conducted every 3 months for the first 2 years following treatment, every 6 months for the next 3 years, and annually after the initial 5 years.

### Statistical analysis

OS was calculated from the time of diagnosis until death from any cause or until the time of the last follow-up visit by the surviving patients. PFS was defined as the interval from the initiation of chemotherapy to the time of the first documented disease progression or recurrence, or from chemotherapy initiation to the last follow-up visit. The Chi-square test was used to calculate statistical group comparisons of categorical variables. Survival analysis was performed using the Kaplan-Meier method, with comparisons made using the log-rank test. Multivariate analysis with a Cox regression model was used to estimate the prognostic impact of different variables on OS and PFS. The variables that were included in a multivariate analysis were statistically significant in univariate analyses. Values of *P* < 0.05 was considered significant, and all *P*-values corresponded to two-sided tests. All statistical analyses were performed using SPSS 16.0 software.

## SUPPLEMENTARY MATERIALS TABLE


