# Supplementary material for: A childhood chemotherapy protocol improves overall survival among adults with T-lymphoblastic lymphoma
Source: Oncotarget. 2016 May 2;7(25):38884–91. doi: 10.18632/oncotarget.9144 (PMC5122438; doi:10.18632/oncotarget.9144)
Supplement: Supplementary file 1 [file oncotarget-07-38884-s001.pdf]

A childhood chemotherapy protocol improves overall survival among adults with T-lymphoblastic lymphoma

Supplementary Materials

Supplementary Table S1: The comparison of baseline clinical characteristics of patients between child and adult regimens

Clinical Characteristics	Childhood regimen NO. of patients (%)	Adult regimen NO. of patients (%)	*P*-value[a]
**NO. of patients**	68 (61.8)	42 (38.2)	
Age			0.468
< 40	56 (82.4)	32 (76.2)	
≥ 40	12 (17.6)	10 (23.8)	
Gender			0.139
Male	44 (64.7)	33 (78.6)	
Female	24 (35.3)	9 (21.4)	
ECOG PS			0.365
≤ 1	49 (72.1)	34 (80.1)	
≥ 2	19 (27.9)	8 (19.9)	
Ann Arbor stage			0.309
I/II	10 (14.7)	10 (23.8)	
III/IV	58 (85.3)	32 (76.2)	
B symptoms			0.422
Present	29 (42.6)	14 (33.3)	
Absent	39 (57.4)	28 (66.7)	
Mediastinal mass			0.428
Present	59 (86.8)	34 (81.0)	
Absent	9 (13.2)	8 (19.0)	
BM involvement			0.238
Positive	41 (60.3)	20 (47.6)	
Negative	27 (39.7)	22 (52.4)	
CNS involvement			1.000
Positive	1 (1.5)	1 (2.4)	
Negative	67 (98.5)	41 (97.6)	
LDH[a]			0.126
≤ 245	32 (50.0)	22 (68.8)	
> 245	32 (50.0)	10 (31.2)	
IPI			
≤ 1	37 (57.8)	22 (68.8)	0.376
≥ 2	27 (42.2)	10 (31.2)	

[a]Data of LDH were available for 96 patients.

Abbreviations:ECOG PS, Eastern Cooperative Group performance status; BM,bone marrow; CNS, central nervous system; LDH, lactatedehydrogenase; B symptoms, tumor fever higher than 38°C, night sweats, and/or weight loss more than 10%; T-LBL,T-lymphoblastic lymphoma.
